# An Unusual Case of Acute Epstein-Barr Virus Hepatitis Presenting as Severe Cholestatic Liver Disease Inducing Hemophagocytic Lymphohistiocytosis in a Young Adult: A Case Report

**DOI:** 10.7759/cureus.87066

**Published:** 2025-06-30

**Authors:** Harpreet S Dosanjh, Primala K Dosanjh, Harendra Ipalawatte, Zain Mehdi

**Affiliations:** 1 Internal Medicine, Los Robles Regional Medical Center, Thousand Oaks, USA

**Keywords:** acute ebv hepatitis, epstein-barr, hemophagocytic lymphohistiocytosis, infectious disease, n-acetylcysteine

## Abstract

Epstein-Barr virus (EBV) infection is a common viral illness typically presenting with symptoms such as fever, sore throat, and lymphadenopathy. Hepatic involvement in EBV infection is usually mild and transient. However, severe cholestatic liver disease due to acute EBV hepatitis is rare, especially in young adults. Secondly, hemophagocytic lymphohistiocytosis (HLH), the abnormal activity of lymphocyte function leading to hemophagocytosis and multi-organ failure, is a rare complication of EBV. In the context of Los Angeles County, the locale of this study, the incidence of secondary nonfamilial HLH among patients over the age of 15 is reported at 0.9 cases per million annually, with epidemiological data specific to EBV-associated HLH even more notably limited. We report a case of a 20-year-old female patient presenting with fever and chills, ultimately diagnosed with acute EBV hepatitis causing severe cholestatic liver injury, with concurrent positive antimitochondrial antibody and HLH, without multi-organ failure, who showed significant improvement with the administration of N-acetylcysteine (NAC), highlighting its potential therapeutic role in EBV-associated liver diseases.

## Introduction

Epstein-Barr virus (EBV) in the Orthoherpesviridae family, also known as human herpesvirus 4, is a double-stranded DNA virus, recognized as one of the most common human viruses worldwide. Primary infection with EBV typically results in a benign, self-limited illness such as infectious mononucleosis, characterized by fever, pharyngitis, and lymphadenopathy. Mild hepatic involvement, evidenced by transient elevations in aminotransferases, is frequently observed. However, the development of severe cholestatic hepatitis secondary to EBV is exceedingly uncommon, particularly among young, otherwise healthy adults.

In addition to hepatic manifestations, EBV is implicated in the pathogenesis of hemophagocytic lymphohistiocytosis (HLH), a rare and often fatal hyperinflammatory syndrome marked by excessive activation of macrophages and cytotoxic T-cells. EBV-associated HLH poses significant diagnostic and therapeutic challenges, given its nonspecific presentation and rapid clinical deterioration if not promptly recognized.

Here, we describe a rare case of a 20-year-old female patient who presented with acute EBV hepatitis resulting in severe cholestatic liver injury, complicated by the development of HLH without multi-organ failure. The patient demonstrated significant clinical improvement following treatment with N-acetylcysteine (NAC), suggesting a potential therapeutic benefit of NAC in managing EBV-associated hepatic dysfunction. This case highlights the importance of maintaining a high index of suspicion for EBV-induced HLH and supports further exploration of NAC as an adjunctive therapy in EBV-related liver injury.

## Case presentation

A 20-year-old female patient with a past medical history of polycystic ovarian syndrome presented to the emergency department with a one-week history of fever and chills, after multiple outpatient diagnoses, including strep throat and otitis media. The patient was initially prescribed amoxicillin for suspected strep throat. However, due to relentless fever and chills, amoxicillin was escalated to Augmentin due to concern for otitis media. After completion of two days of amoxicillin and three days of Augmentin, she then presented to the emergency department with recurrent fever and chills, with recorded at-home temperatures as high as 39.4°C. She denied any recent travel or sick contacts. Outpatient monospot, COVID, and influenza tests were negative. 

Upon presentation, the patient’s initial temperature was 38.4°C with a heart rate of 113 beats/minute, a respiratory rate of 20 breaths/minute, a blood pressure of 114/70 mmHg, and an oxygen saturation of 100% on room air. On physical exam, the patient was mildly jaundiced with right upper quadrant tenderness to deep palpation. Given her presentation and outpatient course, she was empirically given 1 gram of ceftriaxone in the emergency department. Blood cultures and urine cultures were collected. CT of the abdomen and pelvis with IV contrast in the emergency department was obtained and indicated a contracted gallbladder, splenomegaly, and mildly enlarged liver.

Initial laboratory findings were significant for white blood cells of 7.0 x 103/ul (neutrophils, 55.4%), thrombocytopenia 138 x 103/ul, and hemoglobin of 13.7, with very strong agglutinins present on complete blood count (cold agglutinins are autoantibodies typically IgM that bind to red blood cells (RBCs) at lower temperatures, usually below core body temperature. These antibodies can cause RBC agglutination and subsequent hemolysis, particularly in colder peripheral circulation. Laboratory results also indicated hyponatremia and hypokalemia with elevated creatinine, in alignment with hypovolemia from poor oral intake and vomiting. Comprehensive metabolic panel was also remarkable for elevated total and direct bilirubin, aspartate aminotransferase, alanine transaminase, and alkaline phosphatase. Chest X-ray did not indicate any signs of any consolidations or other cardiopulmonary pathology. Blood and urine cultures were negative. Given the presence of cold agglutinins, the emergency department consulted hematology/oncology, who initiated a full autoimmune and malignancy workup, including complement, antinuclear antibody (ANA), C3/C4, hemochromatosis panel, and antismooth muscle, which were all negative. Antimitochondrial antibody was found to be positive but was determined by gastroenterology and hematology to be a false positive and was rechecked outpatient. 

**Figure 1:**
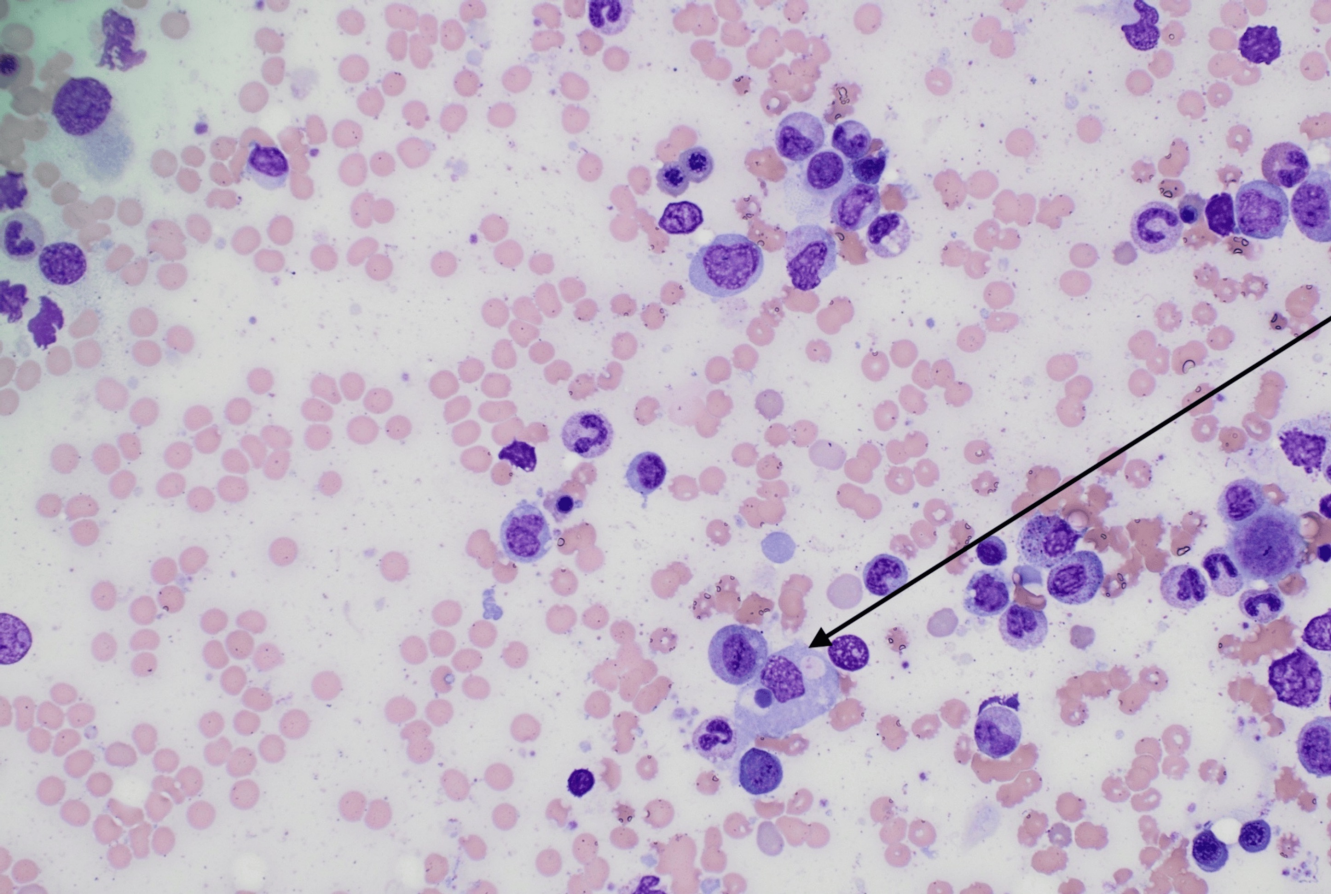
Bone marrow biopsy demonstrating a macrophage with phagocytized lymphocyte

**Figure 2:**
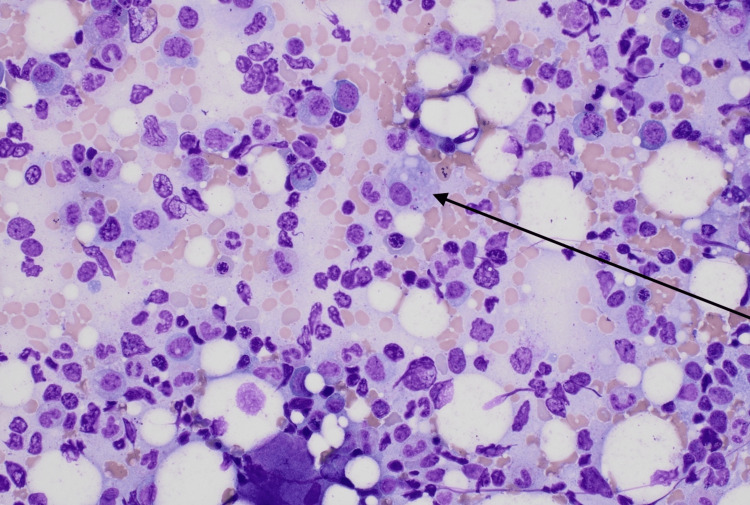
Bone marrow biopsy indicating a macrophage with erythrophagocytosis

Additional laboratory values and units are included in Table 1. Bone marrow findings of Epstein-Barr encoding region-in situ hybridization (EBER ISH)-positive lymphoid cells demonstrating EBV infection and hemophagocytosis (Figures 1, 2), with CD25 greater than 2400, were definitive for HLH. 

**Table 1:** Laboratory value and reference values EBV VCA IGM: Epstein-Barr virus viral capsid antigen IgM

Laboratory tests	Patient laboratory values	Reference laboratory values
Hemoglobin, g/dL	13.7	11.7-15.7
White blood cell	7.0	4.0-11.0
Platelet	138	150-400
Sodium, mmol/L	131	136-145
Potassium, mmol/L	3.4	3.6-5.1
Bicarbonate, mmol/L	21	21-32
Creatinine, mg/dL	1.24	0.55-1.02
Alkaline phosphatase, units/L	269	45-117
Aspartate aminotransferase, units/L	499	10-37
Alanine transaminase, units/L	514	13-56
Total bilirubin, mg/dL	6.0	0.2-1.0
Bilirubin direct, mg/dL	4.19	<0.2
EBV VCA IGM, U/mL	70.3	0.0-35.9
Ferritin, ng/mL	3057.3	8-252
Triglycerides, mg/dL	188	<150
Hepatitis acute panel	Negative	Reactive

The patient was diagnosed with acute EBV hepatitis via an elevated EBV IgM greater than double the upper limit of normal, causing severe cholestatic liver disease. The patient was started on supportive measures, including intravenous hydration and pain management. Given the severity of her liver injury, treatment with NAC was initiated due to its antioxidant and hepatoprotective properties [1,2]. The patient showed remarkable improvement in liver function tests and resolution of symptoms within five days of NAC therapy. Bone marrow biopsy was also obtained due to concerns of HLH. The results of which included EBER ISH-positive lymphoid cells, demonstrating EBV infection, as well as morphologically identified hemophagocytosis, diagnostic for HLH, which included findings of hemophagocytosis, fever, splenomegaly, ferritin >500 microgram/L, and soluble CD25 (IL-2 receptor alpha) greater than 2400 U/ml (4824 U/ml), which are all noted in the criteria. The initiation of rituximab therapy, typically indicated to improve symptoms, viral load, and inflammation associated with HLH, was held due to the patient’s stable clinical presentation [3]. The patient remained stable with close monitoring over a course of 2-3 days, and a follow-up with hematology/oncology outpatient was arranged.

## Discussion

Acute EBV hepatitis presenting as severe cholestatic liver disease is a rare manifestation of EBV infection, especially in young adults. The mechanism of liver injury in EBV infection is not fully understood but is thought to involve direct viral cytopathic effects and immune-mediated hepatocellular damage [4]. The use of NAC in the management of acute liver injury, including drug-induced liver injury and viral hepatitis, has been well-documented. NAC acts as a precursor of glutathione, a crucial antioxidant in hepatocyte protection against oxidative stress and inflammation. NAC replenishes intracellular glutathione levels, thereby enhancing the cellular response to oxidative stress [5,6]. The rationale for using NAC in EBV infection stems from its potential to alter the immune response and lessen oxidative damage, which is a recognized contributor to the pathogenesis of viral infections. In the setting of EBV infection, oxidative stress may increase the cytotoxicity and inflammatory response of the virus, leading to tissue damage and prolonged illness. By decreasing oxidative stress, NAC can theoretically reduce tissue damage and improve clinical outcomes. Additionally, NAC’s role in altering the immune responses might help in controlling the dysregulated immune activation observed in severe EBV infections [7].

Although EBV infections typically present as asymptomatic or infectious mononucleosis, a significant potential complication of EBV is its association with HLH [8]. HLH is a life-threatening hyperinflammatory syndrome characterized by excessive immune activation and tissue infiltration by activated macrophages and lymphocytes, leading to severe organ dysfunction [9]. In EBV-associated HLH, the virus exacerbates an already dysregulated immune system, especially in individuals with genetic predispositions or underlying immune defects. The diagnosis of HLH is often based on clinical criteria established by the HLH-2004 protocol, which includes molecular markers and clinical features [10]. In our case, the patient exhibited some classic findings, including recurrent fevers, hepatosplenomegaly, elevated ferritin levels, and high soluble CD25 levels, as well as more diagnostic findings seen on bone marrow biopsy. The EBV infection was confirmed through positive EBV DNA in the blood, which was re-run due to higher sensitivity and due to high false negatives with monospot testing early in the disease finding. Findings on bone marrow biopsy, combined with the clinical presentation, strongly suggested EBV-associated HLH. The management of EBV-associated HLH is challenging due to the drastically varied presentations of most patients. In our case, per hematology-oncology’s evaluation, the patient’s presentation was stable for simple monitoring. In other cases, rituximab, a monoclonal antibody against CD20, has shown efficacy in reducing EBV-infected B cells and is increasingly used in EBV-HLH [3]. Additionally, antiviral agents like acyclovir may be considered, although their role is adjunctive due to the predominant immune-mediated pathogenesis of HLH [11].

The prognosis of EBV-associated HLH is variable and depends on the timeliness of diagnosis and the response to treatment. Early and aggressive management can significantly improve outcomes, but the condition remains associated with high morbidity and mortality [8]. Long-term follow-up is essential to monitor for recurrence and manage potential complications. As discussed above in the presented case, the patient remained stable without any adjunct treatment for EBV-induced HLH, and her cholestatic injury responded well to NAC therapy. Due to her unusual presentation of EBV-induced HLH, as well as severe cholestatic profile, the patient will require continued monitoring to ensure sustained remission and to address any long-term sequelae of the disease process. However, the positive antimitochondrial antibody, which was found to be positive, was determined to be a false positive due to the presence of the EBV virus and was rechecked outpatient to be negative. 

## Conclusions

This case underscores the importance of considering acute EBV infection as a potential etiology in patients presenting with severe cholestatic liver disease, particularly in the absence of other identifiable causes. Early recognition and initiation of appropriate therapy, including supportive measures and NAC administration, can lead to rapid clinical improvement and favorable outcomes in such patients. Further studies are warranted to elucidate the pathophysiology of EBV-associated liver injury and evaluate the role of NAC in its management. NAC’s well-established safety profile and its antioxidant properties make it a viable candidate for further investigation in the treatment of EBV and other viral infections. This case highlights the complex interplay between EBV infection and the development of HLH, emphasizing the need for higher clinical suspicion and prompt intervention.
